# Primary Squamous Cell Carcinoma of the Breast during Pregnancy: A Case Report

**DOI:** 10.1155/2011/327029

**Published:** 2011-12-21

**Authors:** John Adi Ashindoitiang, Francis Adedayo Faduyile, Olufemi Joshua Taiwo

**Affiliations:** ^1^Surgery Department, General Hospital Ikorodu, Lagos 80112, Nigeria; ^2^Lagos State University Teaching Hospital, Ikeja 21005, Nigeria; ^3^University of Ado Teaching Hospital, Ado Ekiti 2341, Nigeria

## Abstract

Primary squamous cell carcinoma of the breast (SCCB) is a very rare malignancy of the breast and is generally aggressive. It is even rarer during the gestational period. Only few cases have been reported during pregnancy and lactation (Rokutanda et al., 2000). SCCB seen within the gestational period tends to be very aggressive and has a larger size than other breast carcinomas. Pure SCCB is derived from the epidermis of the breast, nipple, or metaplasia on chronic inflammatory background (Bige et al., 2007), such as complicated breast cyst, dermoid cyst, or abscess. We report a case of SCCB in a 30-year-old primigravida that had an aggressive propensity and fatal outcome.

## 1. Introduction

There are small series of report of squamous cell carcinoma of the breast during gestational period in the literature [1]. Most of the other reports of SCCB are in elderly women or atypical presentation following chronic breast pathology. These reported incidences of SCCB vary from 0.1% to less than 0.04% of breast cancers [2–5].

Breast cancer is rare in pregnancy and lactation and varies from 0.4 to 5.0% in western countries [6, 7] and, reported to be 0.4%–1.3% in Japan [8]. We are not aware of the incidence in Africa, and this may be the first reported case of SCCB in pregnancy from Nigeria.

Breast cancer during pregnancy often has poor prognosis, and SCCB is expected to be more aggressive as seen in this reported case in which the patient died within 5 months of diagnosis.

## 2. Case Report

Mrs. S. B., a 30-year-old primigravida at 16-week gestation, presented with a month history of a small polypoid growth on the outer upper quadrant of the left breast. She says it started as a small boil which was progressive. Initially, there was no pain, but later she complained of pains which she rated as 4/10. No prior history of breast lump no nipple discharge existed. She observed that the *polypoid growth bleeds easily on contact. Past medical history revealed no* significant findings. There was no known allergic history, and she was not on any medications except for routine antenatal vitamins supplement and calcium. Her obstetric and gynecological history revealed a primigravida at 16-week gestation; There is no history of oral contraceptives. There was a positive first-degree family history of breast cancer (sister) who died at 28 years after 8 months of diagnosis although the histology was not known. Patient does drink alcohol and does not smoke. She was sexually with one partner, her spouse. Physical examination revealed a young woman in reasonable stable state of health. Abdominal examination revealed uterine size consistent with date. Breast examination showed the right breast to be grossly normal, but the left breast has polypoid growth on the upper outer quadrant that was contiguous with 7 cm in diameter breast lump. A diagnosis of pyogenic granuloma to rule out breast cancer in pregnancy was made. She was evaluated with complete blood count (CBC), ESR, Urea and Creatinine, HIV serology, and later excision biopsy. All investigations were within normal limit except the histopathology of the breast mass which showed invasive squamous cell carcinoma (large cell type) (photomicrographs below).

She was counseled for radical mastectomy since she presented at second trimester, but the patient declined and was lost to followup.

**Figure 1:**
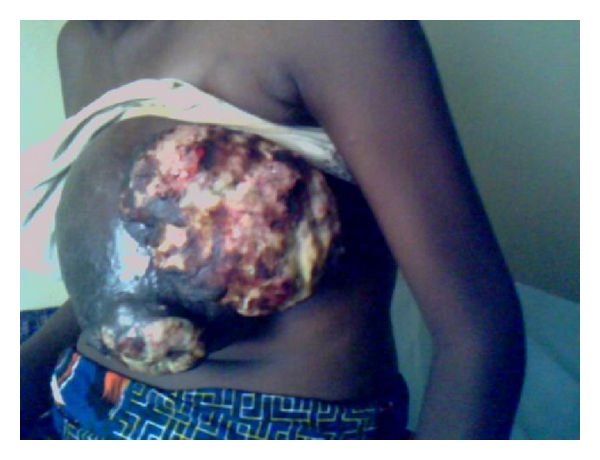
Showing extensively fungating and ulcerated breast lesion.

She later represented at 35 weeks of gestation, and clinical reevaluation revealed an ill-looking woman, with massive fungating, ulcerated, and grossly septic left breast lesion (Figure 1). There was no axillary lymph node involvement. She was admitted for comanagement with the obstetrician. A week later, she delivered a 2.5 kg baby girl. Two days postpartum while treatment options have been considered, she passed away.

## 3. Discussion

Squamous cell cancer of the breast (SCCB) is extremely a rare malignancy. The diagnosis of primary squamous cell cancer is made when the malignant cells are entirely of squamous type and the adjoining breast malignancy or other sites of squamous cell cancer have been excluded [5]. These tumors are reported to be aggressive and have poor prognosis. The prognosis is even worse when this rare tumor is found in the gestational period. Previous report by Rokutanda et al. [8] observed that even when gestational SCCB was diagnosed early and treated, the outcome was still poor.

Our patient declined treatment, and hence, the deterioration was rapid and fatal.

Rokutanda et al. [8] also reported that breast tumors during gestational period tend to be very large, and this was observed in this patient within 4 months of diagnosis (Figure 1).

We could not obtain mammography in this patient to demonstrate the tumor within the breast because of her pregnancy, and ultrasonography was not necessary as there was extension to the skin. Furthermore, SCCB does not have characteristic mammographic features. Some tumors have been reported to have irregular indistinct borders or to be circumscribed.

It was also observed that despite the aggressive nature of the disease in our patient, there were no axillary lymph nodes metastases.

**Figure 2:**
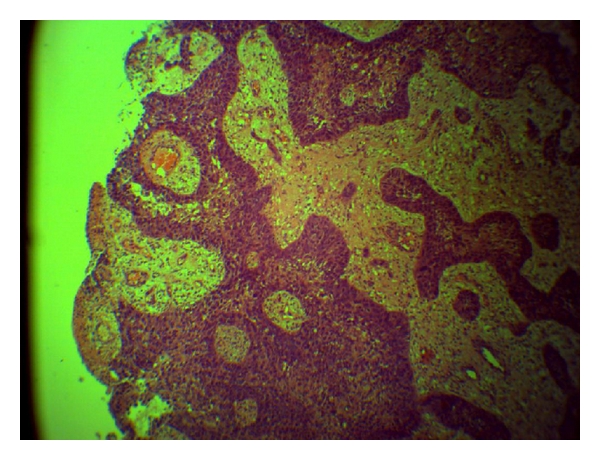
Photomicrograph of the breast lesion (×100). Section shows sheets and cords of malignant squamous epithelial cells invading the fibrocollagenous stroma. These sheets are of variable sizes and show focal areas of necrosis as well as granulation tissue formation. The stroma shows extensive desmoplasia with moderate infiltration by acute and chronic inflammatory cells. No keratin pearl is seen.

**Figure 3:**
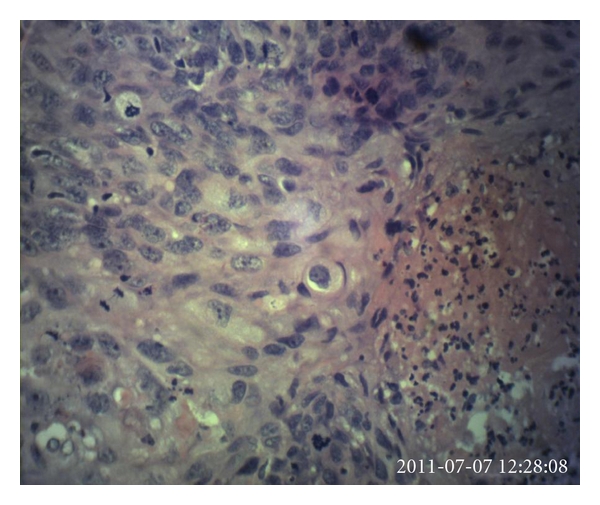
Photomicrograph of the breast lesion at higher power (×400). Section shows malignant squamous cells having large, highly pleomorphic vesicular to basophilic nuclei with conspicuous nucleoli. Many mitotic figures are seen on this field. Area showing suppurative necrosis is seen on the right lower corner. No keratin pearl is seen.

 There are histopathological criteria for diagnosis of primary squamous cell carcinoma of the breast. These criteria include (1) greater than 90% of malignant cells of squamous origin (Figures 2 and 3), (2) tumor independent of the overlying skin and the nipple (nipple is still not involved as this case advanced), and (3) other sites of primary squamous cell carcinoma are excluded [9, 10].

The pathogenesis of SCCB is unclear. Theories include malignant growth of intrinsic epidermal elements (epidermal or desmoids cyst) or metaplasia from breast parenchyma diseases like from cystosarcoma phyllodes, fibroadenoma, or breast malignancy like intraductal cancers or from chronic breast abscess [11].

The World Health Organization categorised these tumors as metaplasia carcinoma [10, 12].

There may be a genetic implication in the etiology of this tumor. This is speculated because the sister of our patient died at early age because of breast cancer. Although we did not know the histology of the sister's breast lesion, this patient's disease may have relationship with her sister as both sisters died within the same age range. A mutant gene may be involved in the etiology of SCCB.

The management of SCCB is radical mastectomy and adjuvant radiotherapy and chemotherapy. The patient refused surgery, and we could not offer her either radiotherapy or chemotherapy because of her pregnancy.

The short period from diagnosis to her death demonstrates that this tumor is really aggressive and more so during gestational period.

## 4. Conclusion

Squamous cell carcinoma of the breast is a rare tumor and is even extremely rare during gestational period. Most of SCCB is diagnosed in elderly women or those with chronic breast lesion. When this tumor is found in young women and gestational period, they are very aggressive and resistant to therapy.
